# Comparing Regenerative and Rehabilitative Strategies for Female Stress Urinary Incontinence: Platelet-Rich Plasma vs. Pelvic Floor Muscle Training—A Prospective Study Evaluating Quality of Life

**DOI:** 10.3390/bioengineering13020242

**Published:** 2026-02-19

**Authors:** Andreea Borislavschi, Cristian-Valentin Toma, Răzvan-Cosmin Petca, Răzvan Dănău, Aida Petca

**Affiliations:** 1 Department of Obstetrics and Gynecology, “Carol Davila” University of Medicine and Pharmacy, 8 Eroii Sanitari Blvd., 050474 Bucharest, Romania; andreea.borislavschi@drd.umfcd.ro (A.B.); aida.petca@umfcd.ro (A.P.); 2 Department of Obstetrics and Gynecology, Elias University Emergency Hospital, 17 Mărăști Blvd., 050474 Bucharest, Romania; 3 Department of Urology, “Carol Davila” University of Medicine and Pharmacy, 8 Eroii Sanitari Blvd., 050474 Bucharest, Romania; cristian.toma@umfcd.ro; 4 Department of Urology, “Prof. Dr. Th. Burghele” Clinical Hospital, 20 Panduri Street, 050659 Bucharest, Romania; 5 Department of Obstetrics and Gynecology, General Hospital CF Number 2, 63 Mărăști Blvd., 011163 Bucharest, Romania

**Keywords:** stress urinary incontinence, PRP, PFMT, gynecology, regenerative medicine

## Abstract

Background: Stress urinary incontinence (SUI) is one of the most common pelvic floor disorders in women, often impairing quality of life (QoL). Pelvic floor muscle training (PFMT) is the standard conservative therapy, while autologous platelet-rich plasma (PRP) is a newer minimally invasive regenerative option. Objective: To compare the effectiveness of three periurethral PRP injections versus PFMT in women with SUI. Methods: This prospective cohort study included 169 women diagnosed with SUI, divided into a PRP group (n = 131), receiving three periurethral PRP injections at 4–6-month intervals, and a PFMT group (n = 38), completing a 12-week PFMT program. Outcomes were measured using the Stamey incontinence scale, visual analogue scale (VAS), and the King’s Health Questionnaire (KHQ). Results: At baseline, PRP patients had more severe symptoms and worse QoL scores. After one injection, PRP achieved improvements in Stamey and VAS scores comparable to PFMT (lower scores), though KHQ remained superior in PFMT (significantly higher baseline scores in the PRP group than the PFMT group). The PRP group showed consistently larger within-group improvements across all scales (*p* < 0.001), in contrast to the PFMT group, which produced smaller and less consistent changes. Conclusions: Repeated PRP treatment provides greater, controlled, and more consistent benefits than PFMT for SUI.

## 1. Introduction

Stress urinary incontinence (SUI), defined as the involuntary loss of urine during increases in intra-abdominal pressure, is a pathology that inflicts quality of life (QoL) in women [1,2]. In a review encompassing population-based studies from multiple countries, the reported prevalence of urinary incontinence (UI) is between 5% and 70%, with most investigations documenting rates of any UI between 25% and 45%, and is associated with considerable psychosocial burden, including social withdrawal and embarrassment [3,4,5]. The prevalence increases progressively with age, affecting more than 40% of women aged 70 years and older. Rates are even higher among the very elderly and in institutionalized populations, such as nursing home residents [3]. SUI was evaluated in a recent systematic review with ten observational studies comprising 18,245 participants. The pooled prevalence of SUI was 26%. Subgroup analyses demonstrated a prevalence of 17% in the general population and 33% among specific cohorts, including postpartum women and individuals with low back pain [5,6]. Global data indicate that approximately 29% to 75% of women will experience SUI at some stage in their lifetime [6,7].

Major risk factors include pregnancy, vaginal delivery, menopause, obesity, aging, and comorbidities such as diabetes mellitus [6,8,9,10,11].

Conservative management remains the first-line treatment for SUI, with international guidelines that recommend pelvic floor muscle training (PFMT) as the initial therapeutic approach [12]. PFMT adherence is frequently suboptimal, as it depends on patient motivation and correct performance of these exercises. PFMT is designed to enhance muscle endurance, strength, power, and relaxation, either individually or in combination. By improving pelvic floor muscle function, PFMT has been shown to reduce disability and limitations in daily activities, thereby supporting greater social participation. These functional benefits are also associated with improved psychosocial well-being [13].

For patients who do not achieve satisfactory outcomes, surgical options such as mid-urethral slings for women diagnosed with SUI may be considered, but these are associated with potential complications and are not appropriate for all women [14]. Regulatory warnings were raised in several countries due to rising adverse reactions to mid-urethral sling surgical intervention for SUI in women. In the United Kingdom, the Independent Medicines and Medical Devices Safety Review has paused the usage of mesh, and the United States FDA (Food and Drug Administration) recommends them under strict conditions [15,16]. A mid-urethral sling remains the gold-standard surgical treatment for SUI [16].

In recent years, regenerative therapies have been investigated as alternatives to conventional treatments. Platelet-rich plasma (PRP), an autologous preparation enriched with platelets and growth factors, has demonstrated the ability to promote angiogenesis, tissue repair, and neuromuscular regeneration [17,18,19].

Despite its widespread use, there is no consensus on the optimal method of platelet activation, the ideal dose, frequency, or injection site of PRP [17]. One study proposed the DEPA classification (Dose, Efficiency, Purity, Activation), which standardizes PRP reporting by quantifying platelet dose, production efficiency, product purity, and the activation process. This framework underscores the need for transparent reporting, since product characteristics largely depend on the preparation system used rather than physician choice [17,18].

Preliminary clinical studies suggest that PRP injections may reduce urinary leakage and improve QoL in women with SUI, with a favorable safety profile [20,21]. However, most available studies are small, single-center pilot trials or case series, and they frequently lack standardized preparation protocols, adequate control groups, and long-term follow-up [22]. Moreover, the literature on this matter has observed that while platelet count is often reported, its direct relevance to clinical outcome remains uncertain in many PRP applications [23,24].

Literature research is limited in comparing PRP treatment with established conservative therapies. However, to our knowledge, no prospective cohort study has directly compared PRP with PFMT as independent interventions, highlighting an important gap in the knowledge.

The present prospective cohort study was therefore designed to evaluate the comparative effectiveness of PRP injections versus PFMT in women diagnosed with SUI. Outcomes were assessed using the King’s Health Questionnaire (KHQ) [25], a validated disease-specific QoL instrument, a visual analogue scale (VAS) [26], and the Stamey incontinence scale [27] for symptom severity.

## 2. Materials and Methods

We conducted a prospective cohort study at Elias Emergency University Hospital, in the Obstetrics and Gynecology Department, between November 2022 and March 2025. This study was designed to compare the effectiveness of PRP injections and PFMT in women diagnosed with SUI. The protocol was approved by the ethics committee (no. 2449/23 March 2022), written informed consent was obtained from all participants prior to enrollment, and the KHQ was used with permission obtained from Mapi Research Trust through ePROVIDE™ (Lyon, France) and administered in accordance with the terms of User License Agreement No. 120730.

SUI severity was evaluated using the Stamey incontinence score, which classifies incontinence into three grades, with higher grades indicating more severe leakage. Symptom severity was additionally assessed using a VAS, ranging from 0 (no symptoms) to 10 (maximum symptom severity), where higher scores reflect greater subjective symptom burden. Health-related QoL was assessed using the KHQ, a validated condition-specific instrument, in which higher scores indicate greater impairment and poorer QoL [25,26,27].

Women aged 40–89 with a clinical diagnosis of SUI reported by the patient and confirmed by gynecological examination (positive cough test, positive Valsalva stress test) were eligible for inclusion (Table 1). Demographic and clinical characteristics, including age, parity, menopausal status, body mass index (BMI), comorbidities (diabetes mellitus, hypertension), use of tobacco status, and baseline symptom severity, were recorded at baseline.

### 2.1. Intervention

Participants in the PRP group underwent three periurethral injections of autologous PRP at intervals of 4–6 months. A total of 40 mL of autologous peripheral venous blood was collected into sterile tubes containing anticoagulant. Samples were processed homogenously using a single centrifugation cycle at 4000× *g* rpm for 7 min, without the use of a commercial preparation kit (open technique). Following centrifugation, the upper plasma fraction and buffy coat were carefully manually aspirated with a sterile syringe, taking care to avoid disturbance of the underlying red blood cell layer, yielding approximately 5 mL of PRP. Activation of platelets was not used.

**Figure 1:**
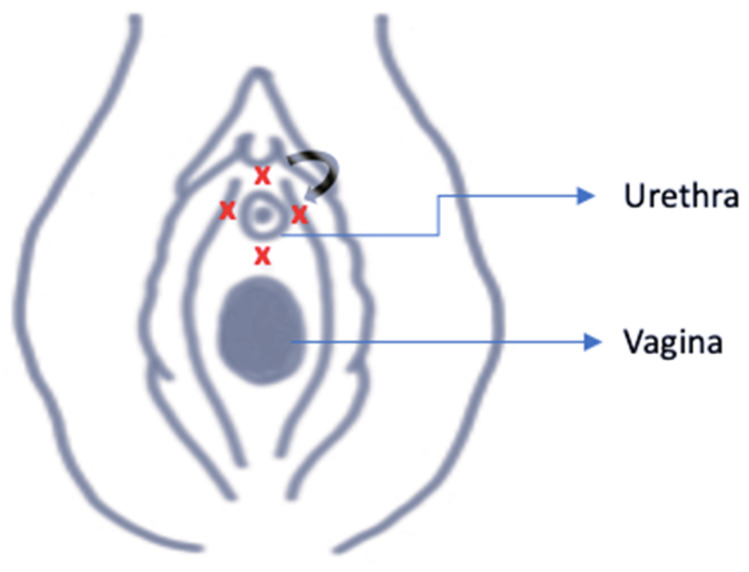
Sites of injection. Schematic representation of the periurethral PRP injection technique. The red “X” marks indicate the injection sites around the urethra, while the arrow illustrates the direction of needle insertion during administration.

Under aseptic conditions, after periurethral preparation with povidone-iodine and local anesthesia (infiltration of 2% lidocaine), PRP was injected: 1 mL at the 12, 3, and 9 o’clock positions and 2 mL at the 6 o’clock position, in close proximity to the urethral lumen, with injections distributed along the functional length of the urethra to ensure homogeneous tissue coverage. A fine-gauge needle was employed to reduce patient discomfort. Patients were monitored briefly following the procedure and discharged the same day. Post-procedure recommendations included abstaining from sexual activity, tampon use, and strenuous physical exercise for 48 h. In addition, patients were instructed to avoid non-steroidal anti-inflammatory drugs (NSAIDs) for at least two weeks before and after treatment and to maintain adequate hydration (30–50 mL/kg body weight). KHQ was administered at baseline along with the VAS and Stamey scale and after the third procedure (KHQ was also administered after each session of PRP) (Figure 1).

In this study, we ensured that the preparation of PRP was performed identically for all participants. Prior to blood collection, a full blood count was obtained for each patient, confirming that the platelet count lay within the normal reference range and that there were no known hematologic disorders or other pathologies likely to affect platelet number or function. Although no individual platelet counts were performed on the actual PRP product for each patient, this was not deemed necessary in our protocol, given the standardized preparation method, the uniform baseline platelet status of participants, and the absence of factors known to affect platelet concentration.

Participants assigned to the PFMT group underwent a structured 12-week PFMT program, based on evidence-based recommendations [12,28]. Training sessions were performed ideally on a daily basis. At initiation, correct technique was verified under supervision by a clinician using digital palpation. The protocol consisted of three daily sets of 8–12 slow, maximal pelvic floor contractions, each sustained for 6–8 s and followed by an equivalent relaxation period. At the end of each set, 3–5 rapid contractions were added. Exercises were carried out in multiple positions, including supine, sitting, and standing, with gradual progression in intensity throughout the training period. This technique is consistent with the detailed PFMT regimen described by Cho and Kim (2021) [29]. Adherence was encouraged through the use of a patient training diary and reinforced during scheduled telephone check-ins (3-week interval) (Supplementary Table S1).

**Figure 2:**
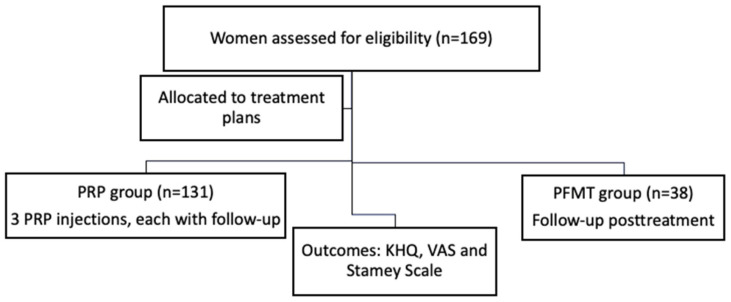
Flowchart of the study.

The study flow is summarized in Figure 2. This study was conducted as a prospective cohort. Treatment allocation was not randomized, as patient preference determined whether participants entered the PRP or PFMT arm, thereby reflecting real-world clinical decision making. This pragmatic approach was chosen to enhance the external validity of this study, mirroring routine clinical practice where patient choice strongly influences therapeutic selection. Another fact we took into consideration when we started this study, as AUA/SUFU Guidelines recommend for patients who seek treatment for SUI, is that the perceived degree of symptom-related bother should be taken into account when selecting an appropriate therapeutic approach [12].

A total of 169 women with stress urinary incontinence were assessed for eligibility and submitted to inclusion and exclusion criteria. After screening, participants were allocated to two treatment groups: 131 women received platelet-rich plasma (PRP) injections (three sessions, with follow-up at post-PRP1, post-PRP2, and post-PRP3), and 38 women underwent pelvic floor muscle training (PFMT/Kegel protocol, with follow-up at post-treatment). Outcomes analyzed included the Stamey incontinence score, visual analogue scale (VAS), and KHQ.

PRP: platelet-rich plasma; n: number; PFMT: pelvic floor muscle training; VAS: visual analogue scale; KHQ: King’s Health Questionnaire.

Data analysis was performed using DATA tab statistical software. Continuous variables were summarized as mean ± standard deviation (SD), while non-normally distributed data were summarized as median with range. Categorical variables were expressed as absolute numbers and percentages. Group comparisons for continuous variables were conducted using independent-sample *t*-tests for normally distributed variables or the Mann–Whitney U test in the case of non-normally distributed variables. Paired comparisons within groups were assessed using the Wilcoxon signed-rank test. Categorical variables were analyzed using the chi-squared test of independence. Effect sizes were calculated (Cohen’s d or correlation coefficient r) to complement significance testing. A two-tailed *p*-value of <0.05 was considered statistically significant.

### 2.2. Outcomes

The primary outcomes represent the improvement in SUI symptoms and the differences between the two groups’ (PRP and PFMT) changes in QoL and symptom severity, measured using the KHQ, Stamey scale, and VAS (baseline and after the three sessions).

Secondary outcomes included: patient satisfaction, complications, and adverse reactions.

Generative artificial intelligence tools (ChatGPT, OpenAI) were used only to assist with phrasing and language refinement during manuscript preparation. No AI tools were used to generate or modify data or influence the scientific interpretation of this study.

## 3. Results

A total of 169 women with SUI were included in this study: 131 received PRP treatment and 38 underwent PFMT. The median age in our cohort was 62.1 years old, with the PRP group having a median of 60.3 years old and the PFMT group having a median of 68.2 years old. The majority of women were postmenopausal in our cohort, with a median duration of 9.2 years in the PRP group and 17.5 years in the PFMT group, indicating that PFMT patients had a longer postmenopausal status (*p* = 0.001).

Arterial hypertensive status differed significantly between groups, with a higher proportion of arterial hypertensive women in the PFMT group compared with the PRP group (65.8% vs. 41.2%, *p* = 0.008). The proportion of smokers did not differ significantly between groups (31.6% vs. 37.4%, *p* = 0.51).

**Figure 3:**
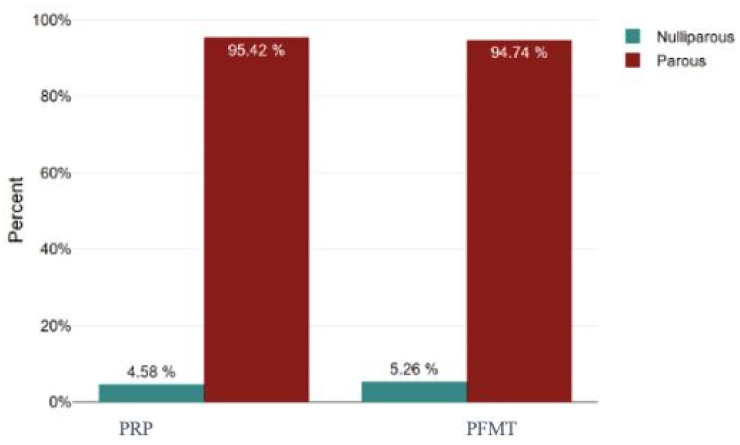
Birth status by subgroup. PRP: platelet-rich plasma; PFMT: pelvic floor muscle training.

Parity (number of births) was similar between groups (*p* = 0.637, Cohen’s d = 0.09), indicating no significant difference. Likewise, body mass index (BMI) did not differ significantly (26.55 ± 3.96 in PRP vs. 27.42 ± 5.49 in PFMT, *p* = 0.33) (Figure 3). The proportion of smokers did not differ significantly between groups (31.6% vs. 37.4%, *p* = 0.51), and the median years of urinary incontinence was 9.2 in the PRP group vs. 19.2 in the PFMT group (*p* < 0.001) (Table 2).

Overall, the two groups were comparable in terms of parity, BMI, and smoking status, while the PFMT group showed a higher prevalence of hypertension.

### 3.1. Stamey Scale

**Figure 4:**
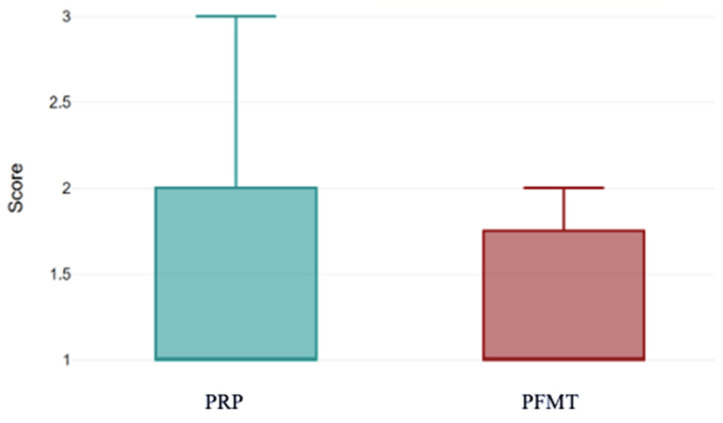
Stamey scale scores pre-treatment for both groups. PRP: platelet-rich plasma; PFMT: pelvic floor muscle training.

At baseline, both groups had a median Stamey score of 1 (PRP group 1.48 ± 0.65 vs. PFMT 1.26 ± 0.45, *p* = 0.085) (Figure 4).

This boxplot compares the distribution of pre-treatment Stamey scores between women who received PRP injections and those who performed PFMT. Median baseline scores are comparable between the two groups, although the PRP cohort demonstrates a slightly wider spread of values, indicating greater variability in initial symptom severity. These data confirm that both groups started from a similar clinical baseline prior to treatment.

**Figure 5:**
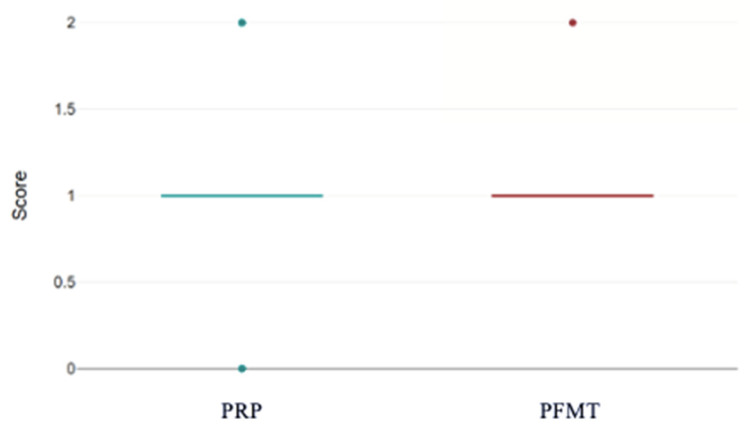
Stamey scale scores post-treatment. PRP: platelet-rich plasma; PFMT: pelvic floor muscle training.

After treatment, both groups demonstrated lower Stamey scores compared with baseline, with no significant difference between PRP and PFMT post-intervention (*p* = 0.427) (Figure 5) but statistically significant improvements within each treatment group (48 ± 0.65 to 1.07 ± 0.3, *p* < 0.001 and 1.26 ± 0.45 to 1.03 ± 0.16, *p* = 0.003, for PFMT).

The figure illustrates the distribution of post-treatment Stamey scores among patients treated with PRP injections and those performing PFMT. Both groups show similar post-intervention median values, indicating comparable improvement in stress urinary incontinence severity following treatment. Individual dots represent the scores recorded for each participant, illustrating the variability within each group. The horizontal line indicates the group mean value, providing a visual comparison of central tendency between PRP and PFMT.

### 3.2. Visual Analogue Scale (VAS) Scores

**Figure 6:**
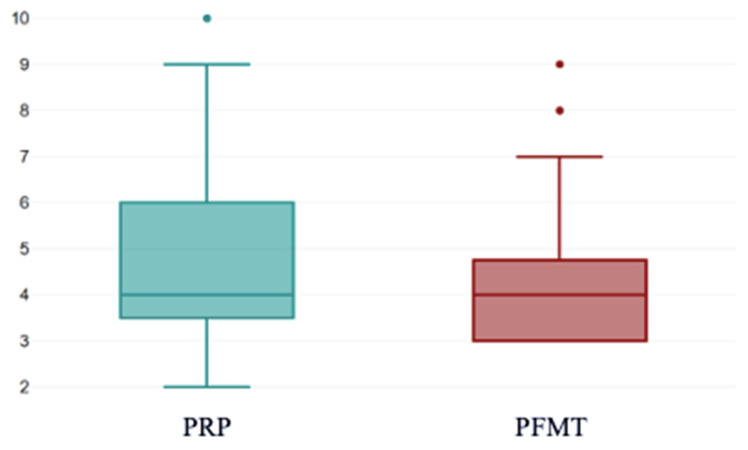
VAS scores pre-treatment. PRP: platelet-rich plasma; PFMT: pelvic floor muscle training; VAS: visual analogue scale.

At baseline, both the PRP and PFMT subgroups had median VAS scores of 4 (mean 4.89 ± 1.85 vs. 4.42 ± 1.85; *p* = 0.059) (Figure 6).

This boxplot displays the distribution of pre-treatment symptom scores among patients assigned to PRP therapy and those allocated to PFMT. Median baseline values were similar between groups, while the PRP cohort exhibited broader variability, reflected by a wider interquartile range and higher outlier values. These findings indicate that both groups entered this study with comparable overall symptom burden, although individual variability was greater in the PRP group The box represents the interquartile range (IQR; 25th–75th percentiles), with the horizontal line inside the box indicating the median. The whiskers extend to the lowest and highest values within 1.5×IQR from the quartiles. Individual dots denote observations outside this range (outliers).

**Figure 7:**
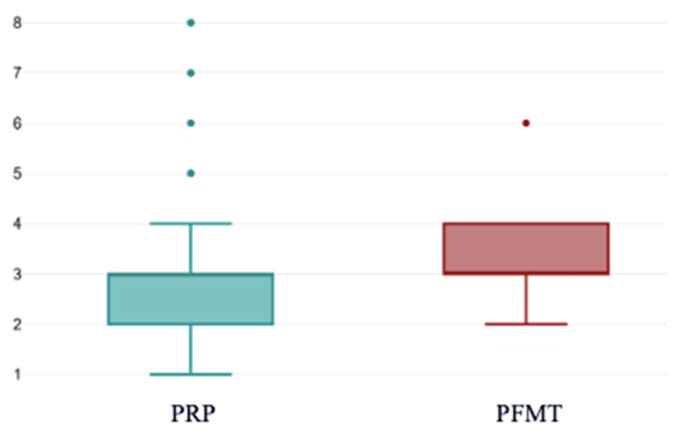
VAS scores post-treatment. PRP: platelet-rich plasma; PFMT: pelvic floor muscle training; VAS: visual analogue scale.

Post-treatment, VAS scores showed a significant decrease compared to the baseline, reaching a median of 3 in both groups (*p* < 0.001 and *p* = 0.002, respectively). However, the PRP group reported significantly lower scores compared to the PFMT counterparts (2.80 ± 1.31 vs. 3.39 ± 0.72 *p* < 0.001) (Figure 7).

This boxplot illustrates the distribution of post-treatment VAS scores in women treated with PRP versus those undergoing PFMT. Median post-treatment pain scores were lower in the PRP group, with a narrower interquartile range, indicating more consistent symptom reduction. In contrast, PFMT showed slightly higher median VAS values and greater variability, suggesting a wider range of individual responses. Several outliers are visible in both groups, reflecting isolated cases with higher residual symptom intensity The box represents the interquartile range (25th–75th percentiles), while the horizontal line within the box indicates the median value. The whiskers extend to the minimum and maximum values within 1.5×IQR. Individual dots represent outliers beyond this range, reflecting greater variability among certain participants.

### 3.3. Quality-of-Life Outcomes (KHQ)

**Figure 8:**
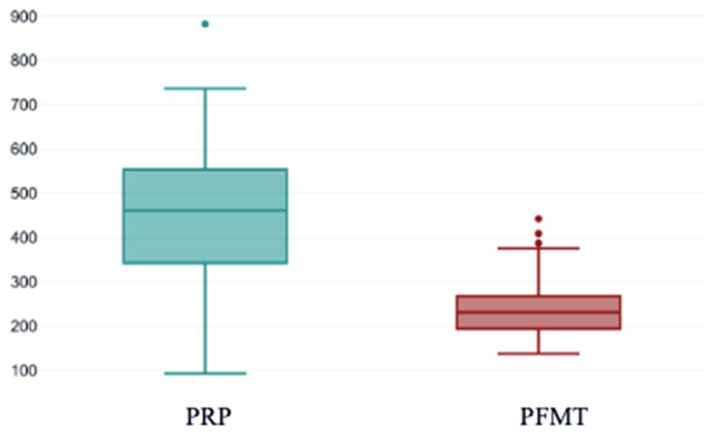
KHQ scores pre-treatment. PRP: platelet-rich plasma; PFMT: pelvic floor muscle training; KHQ: King’s Health Questionnaire.

At baseline, PRP patients reported a higher symptom burden compared to the PFMT groups, with significantly higher KHQ total scores (454.77 ± 142.75 vs. 244.68 ± 75.02; *p* < 0.001) (Figure 8).

This boxplot presents the distribution of pre-treatment KHQ total scores in women allocated to PRP therapy compared with those assigned to pelvic floor muscle training (PFMT). Median baseline KHQ scores were higher in the PRP group, and the interquartile range was broader, indicating greater variability in symptom burden at study entry. In contrast, the PFMT group showed lower and more tightly clustered baseline scores, reflecting a more homogeneous level of QoL impairment prior to intervention. These differences highlight the variability in initial symptom severity between groups before treatment initiation. *The box represents the interquartile range (25th–75th percentiles), and the horizontal line inside the box indicates the median score. The whiskers extend to the minimum and maximum values within 1.5×IQR. Individual dots represent outliers. The distribution illustrates higher baseline KHQ scores in the PRP group compared to the PFMT group, reflecting greater impairment in quality of life.*

**Figure 9:**
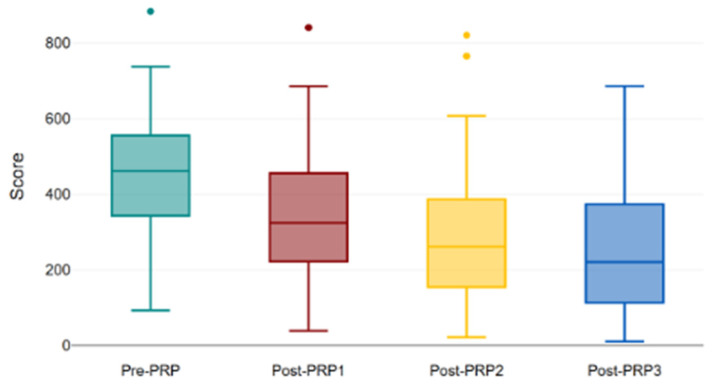
KHQ scores after each injection with PRP.

In the PRP group, the scores progressively improved to a median of 357.8 post-PRP1, 293.4 post-PRP2 and 248.45 post-PRP3. All within-group comparisons were highly significant (*p* < 0.001) (Figure 9).

This boxplot illustrates the progressive reduction in KHQ total scores following PRP therapy. Baseline scores (pre-PRP) were the highest, followed by sequential decreases after the first (post-PRP1), second (post-PRP2), and third (post-PRP3) treatment sessions. The pattern demonstrates a cumulative improvement in symptom burden and QoL throughout the PRP treatment protocol. PRP: platelet-rich plasma. Box-and-whisker plots illustrating the evolution of KHQ total scores following successive PRP treatment sessions (Pre-PRP, Post-PRP1, Post-PRP2, and Post-PRP3). The boxes represent the interquartile range (25th–75th percentiles), with the horizontal line inside each box indicating the median value. The whiskers extend to the minimum and maximum values within 1.5×IQR, while individual dots denote outliers. The progressive downward shift of the median across sessions reflects a sustained improvement in quality of life after repeated PRP administrations.

In the PFMT group, KHQ scores also decreased significantly from 244.68 ± 75.02 to 226.82 ± 70.34 (*p* < 0.001).

**Figure 10:**
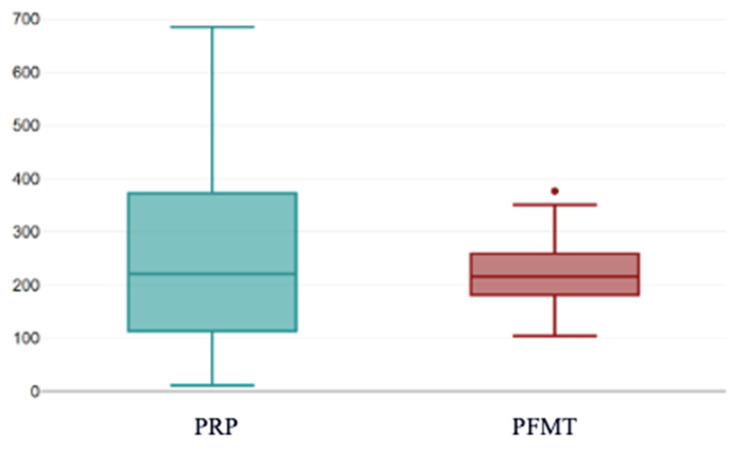
KHQ scores post-treatment. PRP: platelet-rich plasma; PFMT: pelvic floor muscle training; KHQ: King’s Health Questionnaire.

Post-treatment, no significant difference was found between PRP and PFMT (*p* = 0.862) (Figure 10).

This boxplot depicts the distribution of post-treatment KHQ total scores in women treated with PRP compared with those undergoing PFMT. The PRP group shows a wider range of post-treatment scores, with a higher upper whisker, indicating greater variability in residual symptom burden. This finding is consistent with the higher baseline symptom severity and clinical heterogeneity of patients selected for PRP therapy. In contrast, the PFMT group exhibits a more compact distribution with lower median and interquartile values, suggesting a more uniform post-treatment improvement among participants. The presence of outliers in the PFMT group reflects isolated cases with higher post-treatment KHQ impact. In this box-and-whisker plot, the individual dots represent outliers, meaning values that fall outside the expected distribution range (beyond 1.5× the interquartile range from the quartiles).

**Figure 11:**
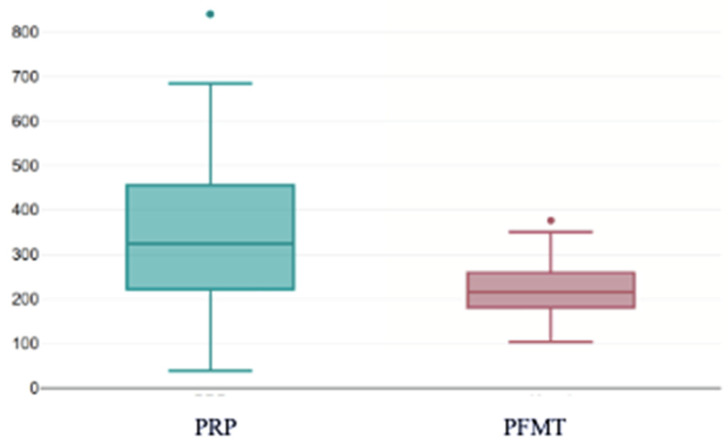
KHQ scores after one injection with PRP compared with scores after PFMT. PRP: platelet-rich plasma; PFMT: pelvic floor muscle training.

After the first PRP injection, the PRP group showed substantially lower KHQ scores compared with PFMT (median 324.00 vs. 215.87), and the between-group difference remained significant (U = 1304, z = −4.46, *p* < 0.001, r = 0.34) (Figure 11).

This boxplot illustrates the distribution of KHQ total scores following a single PRP treatment session, compared with scores in the PFMT group. After one PRP injection, KHQ scores markedly decreased, with substantially lower medians and wider variability compared with PFMT. The difference between the two groups was statistically significant (*p* < 0.001), indicating an early and pronounced improvement in quality of life following the initial PRP session. The individual dots represent outliers, defined as values located beyond 1.5× the interquartile range from the upper or lower quartile. These points indicate observations that deviate substantially from the main distribution of scores within the group.

**Figure 12:**
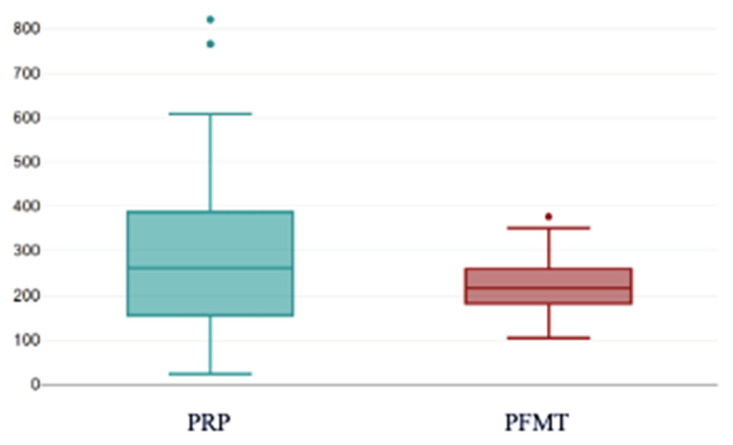
KHQ scores after two injections with PRP compared with scores after PFMT. PRP: platelet-rich plasma; PFMT: pelvic floor muscle training.

At the second post-treatment evaluation (post-PRP2), the median score in the PRP group declined to 260.67, whereas PFMT remained at 215.87. The between-group comparison did not reach statistical significance at this timepoint (U = 2072, z = −1.57, *p* = 0.116) (Figure 12).

This boxplot shows the distribution of KHQ total scores after the second PRP treatment session, relative to the PFMT group. Following two PRP injections, KHQ scores continued to decline, reflecting a sustained improvement in symptom burden and QoL. Although median scores remained lower in the PRP group compared with PFMT, the between-group differences were not statistically significant at this timepoint (*p* = 0.116). *The dots indicate outliers, representing individual values that fall outside 1.5× the interquartile range from the quartiles and therefore lie beyond the whiskers of the boxplot.*

Treatment efficacy was evaluated using the Stamey incontinence score, the VAS, and the KHQ. Both groups demonstrated significant improvements from baseline. However, differences were observed in the magnitude and trajectory of responses. The PRP subgroup showed progressive improvements across repeated injection cycles, while the PFMT subgroup improved after the exercise program but with smaller changes in some domains (Table 3).

Subgroup analyses showed consistent improvement across parity, BMI, and symptom-duration categories, with a more pronounced effect in the PRP group. Among uniparous and multiparous women, PRP produced significant reductions in Stamey, VAS, and KHQ scores (all *p* < 0.001). PFMT also improved symptoms but with smaller effect sizes, achieving significance mainly for VAS and, sporadically, for KHQ. In the overweight subgroup, PRP resulted in significant post-treatment improvements across all three scales, whereas PFMT did not produce significant changes in Stamey or VAS scores. A similar pattern was observed among obese patients, where PRP yielded robust improvements (*p* < 0.001), while PFMT demonstrated only modest changes. Duration-based analyses showed that women with <3 years of SUI experienced the strongest response to PRP, with substantial declines in VAS and KHQ scores. This subgroup contained no PFMT participants, making comparison impossible. Among women with >3 years of SUI, both treatments reduced symptom scores, but PRP consistently demonstrated greater median improvements across all scales, with all comparisons reaching high statistical significance (*p* < 0.001) (Table 4).

No adverse reactions were observed in either the PRP or PFMT groups throughout the study period, confirming the safety of both interventions. Patient-reported satisfaction, however, was notably higher in the PRP group, with women frequently describing greater subjective improvement in continence and QoL. In contrast, although PFMT was effective in reducing symptoms, a considerable proportion of women in this group expressed interest in pursuing PRP therapy in the future, suggesting that while exercise-based therapy is acceptable, the regenerative potential and minimally invasive nature of PRP may be perceived as a more attractive long-term option.

## 4. Discussion

This study compared the effectiveness of PRP injections with PFMT in women with SUI, using three validated measures: the Stamey incontinence score [27], the VAS [26], and the KHQ [25]. Both treatments resulted in significant improvements in continence status, symptom severity, and QoL, but the trajectory and magnitude of improvements differed between groups.

Urinary incontinence severity was assessed using the Stamey incontinence score, a clinical grading system that categorizes SUI into three grades based on symptom severity and circumstances of urine leakage. Symptom intensity was additionally evaluated using a VAS, where patients rated their perceived symptom severity on a continuous scale from 0 (no symptoms) to 10 (maximum severity). Health-related QoL was assessed using the KHQ, a validated, condition-specific instrument designed to evaluate the impact of urinary incontinence on multiple domains of daily life and well-being [25,26,27].

PFMT remains the cornerstone of conservative management for SUI. In this study, a structured 12-week program led to significant improvements in Stamey scores, VAS ratings, and KHQ total scores. These findings are consistent with prior reports demonstrating that correctly performed PFMT enhances urethral support, reduces leakage episodes, and improves patient-reported QoL. The effect sizes in our cohort were moderate, reflecting meaningful but not complete symptom control.

PRP treatment was also associated with significant improvements across all outcome measures. After a single injection, patients reported significant reductions in SUI severity and VAS scores, as well as improved QoL. However, because women in the PRP group started with worse baseline KHQ scores (higher scores), their outcomes after one injection were comparable to those of PFMT patients after 12 weeks of training. The magnitude of improvement from baseline was substantially greater in PRP-treated patients, who had started from a markedly worse QoL profile. This indicates that, despite the apparent absolute difference at follow-up, the relative benefit achieved with PRP after one session was at least comparable, if not superior, to that of PFMT. Taken together with the significant improvements observed in VAS and the comparable reductions in Stamey scores, these findings suggest that a single PRP injection may provide greater overall therapeutic benefit than a standard PFMT program.

Following two injections, PRP patients demonstrated further reductions in KHQ scores, narrowing the gap with PFMT. At this stage, there was no longer a statistically significant difference between the two treatments. By the third injection, PRP patients achieved QoL outcomes that were fully comparable to PFMT, despite their worse baseline impairment. Importantly, PRP showed stronger improvements in symptom perception (VAS) compared with PFMT, suggesting a superior reduction in daily bother.

Taken together, these findings suggest that PRP provides progressive, cumulative benefits; one injection offers results comparable to PFMT in some domains, whereas three injections achieve outcomes equal to or surpassing PFMT, particularly in patient-reported symptom bother. It is important to emphasize that baseline symptoms and QoL scores were substantially higher in the PRP group compared with the PFMT group (Table 2), indicating that patients treated with PRP started from a more severe disease state. This difference is clinically relevant because it places the improvements observed in the PRP group into greater context: despite beginning with worse continence and QoL scores, PRP patients demonstrated significant and progressive improvements that, after repeated injections, reached levels comparable to PFMT. Therefore, the therapeutic effect of PRP may be considered particularly meaningful, as it was able to reduce a higher initial symptom burden to outcomes equivalent to those achieved by PFMT.

These subgroup analyses reinforce the broad efficacy of PRP across diverse patient profiles, suggesting that its regenerative mechanism is effective regardless of parity, body mass index, or chronicity of stress urinary incontinence. Unlike PFMT, which showed variable benefits and limited effect in subgroups with higher risk factors such as obesity or multiparity, PRP consistently produced clinically meaningful improvements. This finding is important, as both obesity and parity are well-established risk factors for SUI and are often associated with suboptimal outcomes following conservative training-based approaches. The robustness of PRP in these more challenging populations highlights its potential as a valuable therapeutic option, particularly for women less likely to achieve satisfactory results with PFMT alone.

PRP serves as an autologous reservoir of signaling molecules. Following platelet activation, α-granules undergo degranulation and release a wide array of growth factors and cytokines that modulate the surrounding microenvironment. Among the principal bioactive molecules released are vascular endothelial growth factor (VEGF), fibroblast growth factor (FGF), platelet-derived growth factor (PDGF), epidermal growth factor (EGF), hepatocyte growth factor (HGF), insulin-like growth factors 1 and 2 (IGF-1, IGF-2), matrix metalloproteinases (MMP-2, MMP-9), and interleukin-8 (IL-8) [17,30,31].

PRP is obtained by centrifugation of the autologous venous blood, which separates its components according to their density. Two main preparation approaches exist [17]:

Open technique: The product is exposed to the environment and requires strict aseptic handling [17].

Closed technique (commercial kits): Minimizes contamination risk [17].

After centrifugation, three layers are visible: red blood cells and leukocytes at the bottom, PRP in the middle, and platelet-poor plasma (PPP) at the top. Platelets may be activated prior to PRP administration; however, there is no consensus regarding whether pre-activation is necessary or which activating agent should be used [17].

PRP demonstrates meaningful efficacy for treating women with SUI, both as a standalone therapy and when it is combined with PFMT [22,32].

Literature review consistently supports PFMT as an effective therapy for enhancing pelvic floor muscle tone and automatic motor control, thereby reducing urine leakage by counteracting increases in intra-abdominal pressure [33]. A systematic review also confirmed overweight and obesity as important risk factors for UI, alongside parity, pregnancy and mode of delivery, smoking, and other comorbidities, as highlighted by the International Continence Society [9,34,35,36,37]. Given these factors, the choice of intervention should be tailored to the patient’s symptoms, degree of bother, expectations, and the balance of risks and benefits. Importantly, women often prefer conservative strategies, with PFMT being recommended as a first-line option for SUI, although it is less effective in cases of urgency urinary incontinence [33].

A recent systematic review challenged the traditional assumption that greater pelvic floor muscle strength directly translates into improved continence outcomes [38]. Although biofeedback-assisted PFMT (BPFMT) was associated with greater gains in muscle strength, the meta-analysis found no significant differences between BPFMT and conventional PFMT in terms of incontinence episodes and daytime or nighttime micturition. Given the higher costs associated with device use, PFMT alone remains a reliable, noninvasive option for improving incontinence symptoms, with outcomes comparable to those achieved with biofeedback [38]. As our study revealed, PFMT showed improved SUI symptoms.

Our results are in line with previous evidence supporting the role of PFMT in improving continence outcomes. A prospective study evaluated the effects of home-based Kegel exercises in 72 women with urodynamically confirmed SUI or MUI (mixed urinary incontinence). After eight weeks of daily unsupervised training, both groups showed significant improvements in pelvic floor muscle strength (Oxford scale), symptom scores (UDI-6, IIQ-7), and patient-reported global impression of improvement, with the greatest benefit observed among women with SUI [39]. Similarly, in our cohort, PFMT led to significant reductions in Stamey and VAS scores and improvements in QoL measured by KHQ. Taken together, these findings reinforce the effectiveness of PFMT as a first-line, low-cost, and noninvasive therapy for SUI. Importantly, our study further extends this evidence by directly comparing PFMT with PRP, highlighting that while PFMT provides reliable benefits, PRP offers a bigger, faster, and controlled improvement. PFMT outcomes are strongly dependent on patient adherence, with consistent practice essential to achieving therapeutic benefit [13,33].

A recent clinical trial evaluated the combination of autologous PRP injections (two injections of PRP in the anterior vaginal wall at a one-month interval) with PFMT in women vs. only PFMT (in the morning, noon, and evening every day, with 8–12 contractions and relaxations per 4–6 s session). The patients were evaluated at 5 months, and the group with PRP and PFMT demonstrated significantly greater reductions in symptoms and higher subjective improvements (90% vs. 14% reporting >50% improvement), compared with the PFMT alone [32]. This study concluded that the reported improvement appears to be mainly due to the administration of PRP [32]. In contrast, our study compared PRP monotherapy with PFMT in women with SUI. After one injection, the group of treatment with PRP, which had significantly worse scores than PFMT, matched PFMT in continence severity (Stamey) and outperformed it in symptom bother (VAS). Although KHQ remained better in PFMT, this reflected worse baseline impairment in the PRP group, highlighting the strong early effect of PRP even in more severely affected patients. Taken together, the published data highlight that combining PRP with PFMT accelerates symptom relief and enhances outcomes, while our findings demonstrate that PRP alone, delivered sequentially, is capable of achieving outcomes comparable to or better than PFMT. This suggests that PRP may act as a standalone regenerative therapy, while its combination with PFMT could further optimize treatment in selected patients.

In our study, patients underwent a series of three periurethral PRP injections, administered at 4–6-month intervals. This protocol contrasts with several published approaches that used either a single injection of PRP or two injections or three injections of PRP in closely spaced sessions [22]. A single anterior vaginal wall (mid-urethral) injection of PRP produced significant improvements in SUI severity at both 1 and 6 months, across multiple patient-reported measures, with no adverse events reported. Treatment response showed a non-significant trend toward greater benefit in younger women, supporting PRP as a safe, promising option for mild-to-moderate SUI [20]. In our study, we used single centrifugation, with non-commercial kits, whereas the literature presents vastly different preparations of PRP, with no standardization regarding preparation [40].

SUI affects both pelvic-floor mechanics and neurobehavioral function, with consequences that extend beyond involuntary leakage to include altered body perception and psychological distress. Parallel progress in gynecologic biomarker research, such as recent advances involving squamous cell carcinoma antigens, supports a more integrated approach to disorders of women’s pelvic health, combining anatomical, neurological, and molecular perspectives [41,42]. Concomitant NSAID therapy was avoided, as they may impair platelet activation and aggregation, potentially reducing the biological activity and regenerative effects of PRP [43,44]. Adequate fluid intake was recommended prior to PRP preparation, as proper hydration is known to influence blood viscosity and platelet concentration [45].

Conventional urethral bulking agents are primarily designed to improve SUI by providing a mechanical volume effect that enhances urethral coaptation, without directly addressing the underlying tissue degeneration. In contrast, PRP represents an autologous biological therapy aimed at promoting tissue regeneration and functional recovery through the release of platelet-derived growth factors and bioactive mediators. Bulking agents’ effects are often transient and may require repeated injections over time, whereas PRP therapy is still studied regarding the long-term effects. Although PRP is not specifically approved by regulatory agencies such as the U.S. Food and Drug Administration (FDA) for the treatment of SUI, some PRP preparation systems are FDA-cleared, and PRP is currently used internationally as an off-label therapeutic option for SUI in clinical and research settings (in our study PRP was prepared using an open technique, without activation) [20,22,46,47,48,49].

The duration of the therapeutic effects of PRP in SUI has not yet been clearly defined. Available studies suggest that PRP may provide symptom improvements lasting several months to over one year, although reported follow-up periods vary considerably. In the present study, beneficial effects were maintained throughout the follow-up period; however, the lack of extended long-term data precludes definitive conclusions regarding durability. Future studies with longer follow-up and repeated outcome assessments are needed to better characterize the persistence of PRP-related effects and to determine optimal re-treatment intervals [21,22].

## 5. Limitations of This Study and Future Perspectives

This study has certain limitations but also notable strengths. Baseline imbalance was present, as women in the PRP group had worse KHQ scores and a shorter duration of SUI at inclusion. While this may introduce the possibility of regression to the mean, it also highlights the robustness of PRP outcomes, since patients starting from a more severe condition were able to improve to levels comparable with PFMT. The non-randomized allocation and unequal group sizes (131 PRP vs. 38 PFMT) reflect real-world clinical practice and ensure feasibility, although they may limit the generalizability of results. While this imbalance may limit the strength of direct comparisons between groups, it reflects routine clinical decision making and highlights PRP as a treatment option predominantly chosen by patients with a higher baseline disease burden.

A limitation of this study is the lack of direct quantification of platelet counts or growth factor concentrations in the PRP used. However, all participants had normal baseline platelet values, no conditions or medications affecting platelet function, and PRP was prepared identically for all patients, reducing variability and strengthening the internal consistency of our findings.

Another limitation concerns the healthcare context in Romania, where PRP therapy in SUI is not covered by public insurance. While evidence-based medicine requires robust data, such evidence cannot accumulate unless the method is applied in sufficiently large and diverse populations. In an era of rising life expectancy, it is essential to establish, beyond reasonable doubt, the clinical value of a treatment that stands out by offering symptom improvement without meaningful adverse effects. Clinically, wider access to PRP could provide a safe option for women whose QoL is significantly impaired by SUI.

Finally, PRP patients were followed longitudinally after each injection, whereas PFMT patients were assessed once post-intervention. This asymmetry in follow-up design should be considered when interpreting temporal comparisons, but it also provided valuable insights into the progressive, cumulative effect of PRP treatment.

## 6. Conclusions

This study demonstrates that periurethral PRP injections are an effective treatment for women with SUI, with progressive and sustained benefits observed after repeated sessions. While PFMT provided meaningful improvements in continence and QoL, a single PRP injection achieved comparable or superior outcomes in symptom severity and patient-reported bother. Importantly, two and three PRP injections produced further significant gains, ultimately surpassing PFMT in most clinical measures, including VAS, Stamey, and KHQ scores. Subgroup analyses confirmed that the therapeutic effect of PRP was consistent across parity, BMI categories, and duration of incontinence, including in women typically considered more difficult to treat. Taken together, these findings support PRP as a safe, regenerative, and effective standalone therapy for SUI, offering an attractive alternative or complement to conservative PFMT.

## Figures and Tables

**Table 1:** Inclusion and exclusion criteria.

Inclusion Criteria	Exclusion Criteria
Stress urinary incontinence reported by the patient and confirmed by gynecological examination Contraindications for surgical treatment Patient’s preference for a non-surgical treatment	Anatomical defect (anterior and/or central compartment prolapse > stage 1)
	Platelet disorders (qualitative or quantitative)
	Sepsis
	Pregnancy
	Chronic use of anticoagulant medication
	Prior anti-SUI surgery
	Neoplasia
	Chronic treatment with NSAIDs
	Refusal to participate

NSAIDs: non-steroidal anti-inflammatory drugs; SUI: stress urinary incontinence.

**Table 2:** Descriptive characteristics of the participants.

Characteristic	PRP Group	PFMT Group	Total Cohort	*p*-Value
Number of participants	131	38	169	-
Mean age (years) ± SD	60.3 ± 11.2	68.2 ± 12.7	62.1 ± 12.2	<0.001
Years since menopause, mean ± SD	9.2 ± 8.6	17.5 ± 13.3	11 ± 10.3	0.001
Number of births, mean ± SD	2.0 ± 0.9	2.1 ± 1.0	2.0 ± 0.9	0.637
Arterial hypertension, n (%)	54 (41.2%)	25 (65.8%)	79 (46.7%)	0.008
Smokers, n (%)	49 (37.4%)	12 (31.6%)	61 (36.1%)	0.51
BMI (kg/m^2^), mean ± SD	26.6 ± 4.0	27.4 ± 5.5	27.0 ± 4.4	0.33
Years of SUI, mean ± SD	9.2 ± 8.6	19.2 ± 11.6	11.4 ± 10.1	<0.001

Continuous variables are presented as mean ± standard deviation. Categorical variables are presented as number and percentage. PRP: platelet-rich plasma; PFMT: pelvic floor muscle training; BMI: body mass index; kg: kilograms; m = meters, SUI: stress urinary incontinence, n: number, SD: standard deviation.

**Table 3:** Comparative outcomes between PRP and PFMT groups.

Outcome	Timepoint	PRP (Mean ± SD)	PFMT (Mean ± SD)	Between-Group *p*-Value
Stamey score	Baseline	1.48 ± 0.65	1.26 ± 0.45	0.085
	Post-treatment	1.07 ± 0.33	1.03 ± 0.16	0.427
VAS score	Baseline	4.89 ± 1.85	4.42 ± 1.85	0.059
	Post-treatment	2.80 ± 1.31	3.39 ± 0.72	<0.001
KHQ total	Baseline	454.77 ± 142.75	244.68 ± 75.02	<0.001
	Post-PRP1/PFMT	357.8 ± 174.03	226.82 ± 70.34	<0.001
	Post-PRP2/PFMT	293.4 ± 183.41	226.82 ± 70.34	0.116
	Post-PRP3/PFMT	248.45 ± 169.59	226.82 ± 70.34	0.862

PRP: platelet-rich plasma; PFMT: pelvic floor muscle training; KHQ: King’s Health Questionnaire; VAS: visual analogue scale.

**Table 4:** Statistical analysis on the stratifications of the subgroups.

Subgroup	Scale	Group	Pre-Median	Post-Median	*p*-Value (Within)
Uniparous	Stamey	PRP	1	1	<0.001
		PFMT	1	1	0.046
	VAS	PRP	5	3	<0.001
		PFMT	4	3	0.027
	KHQ	PRP	495.67	256.67	<0.001
		PFMT	223.42	216.08	0.034
Multiparous	Stamey	PRP	1	1	<0.001
		PFMT	1	1	0.025
	VAS	PRP	4	2	<0.001
		PFMT	3.5	3	0.029
	KHQ	PRP	461.22	226.22	<0.001
		PFMT	238.48	215.86	<0.001
Overweight	Stamey	PRP	1	1	<0.001
		PFMT	1	1	NS
	VAS	PRP	5	3	<0.001
		PFMT	3	3	NS
	KHQ	PRP	482.83	330.22	0.076 (<0.001)
		PFMT	229.92	215.04	NS
Obese	Stamey	PRP	1	1	<0.001
		PFMT	1	1	0.149
	VAS	PRP	5	3	<0.001
		PFMT	4	3	0.026
	KHQ	PRP	493.44	227.89	<0.001
		PFMT	214.97	214.09	NS
<3 years SUI	Stamey	PRP	1	1	0.001
		PFMT	-	-	-
	VAS	PRP	4	2	<0.001
		PFMT	-	-	-
	KHQ	PRP	382.11	80.67	<0.001
		PFMT	-	-	
>3 years SUI	Stamey	PRP	1	1	<0.001
		PFMT	1	1	0.03
	VAS	PRP	5	3	<0.001
		PFMT	4	3	0.02
	KHQ	PRP	464.28	259.67	<0.001
		PFMT	231.97	215.86	<0.001

Notes: Medians reported because outcomes were non-parametric. Within-group *p*-values were obtained using the Wilcoxon signed-rank test for paired pre-/post-treatment comparisons. For KHQ analyses in the PRP group, which included multiple post-treatment timepoints, overall change was evaluated using the Friedman test (chi-squared), with pairwise contrasts applied where indicated. NS = not significant. SUI = stress urinary incontinence; VAS = visual analogue scale; KHQ = King’s Health Questionnaire; PRP = platelet-rich plasma; PFMT = pelvic floor muscle training.

## Data Availability

The data presented in this study are available from the corresponding author upon reasonable request due to privacy and ethical restrictions (GDPR, institutional approval). The King’s Health Questionnaire (KHQ) is licensed and not shareable; access should be requested from Mapi Research Trust (ePROVIDE).

## Supplementary Materials

The following supporting information can be downloaded at: https://www.mdpi.com/article/10.3390/bioengineering13020242/s1; Table S1: Intervention Description Table.
