# Supplementary material for: Comparing Regenerative and Rehabilitative Strategies for Female Stress Urinary Incontinence: Platelet-Rich Plasma vs. Pelvic Floor Muscle Training—A Prospective Study Evaluating Quality of Life
Source: Bioengineering (Basel). 2026 Feb 19;13(2):242. doi: 10.3390/bioengineering13020242 (PMC12938076; doi:10.3390/bioengineering13020242)
Supplement: Supplementary file 1 [file bioengineering-13-00242-s001.zip › bioengineering-4054870-supplementary.pdf]

Supplementary Table S1. Intervention table

Component	PRP Group	PFMT Group
Intervention type	Periurethral injection of PRP	Structured PFMT program
Blood collection	40 mL peripheral venous blood in sterile anticoagulated tubes	-
Processing method	Single centrifugation, 4 000 rpm for 7 min; (buffy coat + plasma aspirated)	-
PRP yield	Approx.5 mL PRP obtained, no commercial kit used	-
Injection protocol	1 mL at 12, 3, and 9 o'clock; 2 mL at 6 o'clock (periurethral)	-
Anesthesia	Local infiltration with 2% lidocaine	-
Post-procedure care	Same-day discharge;<br>advised to avoid NSAIDs (±2 weeks), sex/tampon/strenuous exercise (48h), hydration encouraged	Adherence to program and training diary
Program duration	12-18 months	12 weeks
Frequency	4-6 months	Daily
Exercises	-	8-12 contractions per set, performed in multiple positions
Contraction details	-	6-8 second contraction, equal relaxation time
Supervision	All procedures performed by physician	Technique verified at baseline by digital palpation
Adherence support	Check-ups at every 4-6 months	Training diary and phone check-ins at 3 weeks

PRP: platelet-rich plasma; PFMT: pelvic floor muscle training; RPM: rotations per minute; ml: milliliters; NSAIDs: non steroid anti-inflammatory drugs;
